# Region-Specific and Pregnancy-Enhanced Vasodilator Effects of Hydrogen Sulfide

**DOI:** 10.26502/ogr0145

**Published:** 2023-12-22

**Authors:** Pankaj Yadav, Dong-Bao Chen, Sathish Kumar

**Affiliations:** 1 Department of Comparative Biosciences, School of Veterinary Medicine, University of Wisconsin, Madison, Wisconsin, United States of America; 2 Department of Obstetrics & Gynecology, University of California, Irvine, California, United States of America; 3 Department of Obstetrics and Gynecology, School of Medicine and Public Health, University of Wisconsin, Madison, Wisconsin, United States of America

**Keywords:** H_2_S, artery, vasodilation, pregnant, potassium channels

## Abstract

Hydrogen sulfide (H_2_S) is a cardiovascular signaling molecule that causes vasodilation in vascular smooth muscle cells, but its mechanism is unclear. We examined how H_2_S affects mesenteric and uterine arteries without endothelium in nonpregnant and pregnant rats and the underlying mechanisms. H_2_S donors GYY4137 and NaHS relaxed uterine arteries more than mesenteric arteries in both pregnant and nonpregnant rats. GYY4137 and NaHS caused greater relaxation in the uterine artery of pregnant versus nonpregnant rats. High extracellular K^+^ abolished NaHS relaxation in pregnant uterine arteries, indicating potassium channel involvement. NaHS relaxation was unaffected by voltage-gated potassium channel blockers, reduced by ATP-sensitive potassium channel blockers, and abolished by calcium-activated potassium (BK_Ca_) channel blockers. Thiol-reductant dithiothreitol also prevented NaHS relaxation. Thus, H_2_S has region-specific and pregnancy-enhanced vasodilator effects in the uterine arteries, mainly mediated by BK_Ca_ channels and sulfhydration.

## Introduction

During a normal pregnancy, there is a significant increase in blood flow to the uterus, with a 20–80-fold rise in uterine blood flow observed in the third trimester of a singleton pregnancy [1]. This enhanced uterine perfusion is crucial for maintaining the health of both the mother and the developing fetus as it facilitates the transfer of nutrients and oxygen from the mother to the fetus while also removing carbon dioxide and metabolic waste products from the fetus. Proper uterine blood flow is essential for optimal fetal development and survival, and compromised blood flow has been implicated in various pregnancy-related conditions such as preeclampsia and intrauterine growth restriction [2-5].

The mechanisms governing the dilation of uterine arteries during pregnancy are intricate and not fully understood. However, evidence suggests a pivotal role for locally produced vasodilators in relaxing the smooth muscle of uterine arteries. Among these vasodilators, prostacyclin and nitric oxide (NO) have been extensively studied [6-8]. However, the inhibition of prostaglandin and NO synthesis does not entirely abolish the pregnancy-induced increase in uterine artery blood flow [9-11], suggesting the participation of additional mechanisms. Recent studies indicate an increase in the production of hydrogen sulfide (H_2_S) in uterine arteries during pregnancy, adding it to the family of gaseous signaling molecules that includes NO and carbon monoxide [12, 13]. H_2_S is synthesized from L-cysteine by specific enzymes, and its production in uterine arteries during pregnancy is primarily associated with the upregulation of cystathionine-β-synthase (CBS) [14]. Unlike systemic vasculature, where endothelial cell cystathionine-γ lyase (CSE) is the primary source of H_2_S, uterine artery H_2_S production is linked to both smooth muscle and endothelial CBS upregulation, and this unique H_2_S production is implicated in uterine vasodilation during pregnancy [12, 13]. Studies using a slow-releasing H_2_S donor further support the role of H_2_S as a novel uterine vasodilator [13].

The mechanism by which H_2_S induces uterine artery dilation is not fully understood. Previous research in mesenteric vascular beds has implicated the activation of ATP-sensitive potassium (K_ATP_) channels and large conductance Ca^2+^-activated voltage-dependent potassium (BK_Ca_) channels in mediating H_2_S-induced vasodilation [15-17]. However, the contribution of K_ATP_ channels to uterine vasodilation associated with pregnancy seems limited [15]. In contrast, BK_Ca_ channels have been shown to play a critical role in mediating H_2_S-induced vasodilation in rat mesenteric arteries [18]. This H_2_S-induced mesenteric vasodilation was lost in endothelium-denuded arteries [23], suggesting the presence of endothelium is essential for H_2_S-induced BK_Ca_-mediated mesenteric vasodilation. In contrast, relaxation of the rat aorta and human uterine artery induced by H_2_S appears less dependent on the endothelium [17,19,20], indicating H_2_S directly acts on vascular smooth muscle cells to induce vasodilation. Although BK_Ca_ channels in vascular smooth muscle cells are implicated in pregnancy-induced uterine artery dilation [19,21-23], their involvement in H_2_S-induced vasodilation in rat arteries remains uncertain, and if their sensitivity differs between pregnancy and the nonpregnant state is unknown. Given the recognized heterogeneity in structure, functions, and pharmacological sensitivities of vascular beds, we sought to examine the impact of H_2_S on the vascular relaxation of peripheral resistance arteries, specifically the rat mesenteric and uterine arteries. Since H_2_S is proposed as a therapeutic vasodilator for preeclampsia, wherein the endothelial function is compromised [24-26], it is essential to investigate whether H_2_S directly influences vascular smooth muscle to induce vasodilation. Thus, in this study, we examined the vascular relaxation responses of H_2_S in endothelium-denuded mesenteric and uterine arteries isolated from both pregnant and nonpregnant (virgin) rats and the underlying mechanisms.

## Methods

All the experiments were conducted as per National Institutes of Health guidelines (NIH Publication No. 85–23, revised 1996) with approval by the Institutional Animal Care and Use Committee at the University of Wisconsin at Madison (IACUC protocol V005847). Nonpregnant and timed pregnant Sprague-Dawley rats, purchased from Envigo Laboratories (Indianapolis, IN) on gestation day (GD) 16, were maintained under controlled conditions with a 12L:12D photoperiod in a temperature-regulated room (23°C). These rats were provided with ad libitum food and water. On GD20, pregnant and age-matched nonpregnant rats were euthanized using CO_2_ asphyxiation. Uterine and mesenteric arteries were isolated and immersed in the ice-cold Krebs physiological solution (KPS) (composition-118 mM NaCl, 4.7 mM KCl, 1.2 mM MgSO_4_, 1.2 mM KH_2_PO_4_, 25 mM NaHCO_3_, 11 mM glucose, 0.026mM EDTA, 1.25 mM CaCl_2_; pH 7.4) for study of vascular response.

### Ex-Vivo Vascular Reactivity

Arteries were cleaned from surrounding adventitial tissues, and 1-1.5 mm rings were made, and mounted in wire myograph (Danish Myo Technology, Aarhus, Denmark) using tungsten wires. The bath was filled with 6 ml of KPS continuously aired with carbogen gas (95% O_2_ and 5% CO_2_) and maintained at 37°C. The endothelium was denuded by gently rubbing the interior of the rings with tungsten wire. Rings were equilibrated for 1 hour in KPS and normalized using a specialized software package (Myodata V8.1.13; Danish Myo Technology). Arterial viability and strength were confirmed by depolarizing the rings with KCl 80 mM (two times). The denudation of the endothelial layer was established by the absence of relaxation response to acetylcholine in rings precontracted with a submaximal concentration of phenylephrine (PE). The concentration of PE that induced 80% of the maximal response was utilized for precontraction. Data acquisition was carried out using LabChart software (ADInstruments, Australia).

### Assessment of Vascular Relaxation Responses

The arterial rings were subjected to an 80 mM KCl solution until reproducible contractions were observed. Following a subsequent round of washing and equilibration with KPS, the vascular contractile responses were done by exposing the rings to increasing concentrations of PE (10^−9^ to 10^−5^ M).

The direct effect of H_2_S on vascular smooth muscle relaxation was compared in endothelium-denuded mesenteric and uterine arterial rings of nonpregnant and pregnant rats, using two different H_2_S donors: GYY4137 (a slow-releasing compound; 10^−10^ – 10^−5^ M) and NaHS (a fast-releasing compound; 10^−9^ – 10^−5^ M) in PE precontracted arteries.

### Assessment of H_2_S-induced Vascular Relaxation Pathways

The contribution of potassium channels in the H_2_S-induced vasorelaxation response was assessed by incubating the rings with specific potassium channel blockers, such as 4-aminopyridine (4-AP, voltage-gated potassium channel, 1 mM), glibenclamide (ATP-sensitive potassium channel, 10 μM), iberiotoxin plus apamin (large and intermediate conductance BK_Ca_, potassium channels, 100 nM each), KCl (membrane depolarizer, 80 mM) for 20 min and then examining H_2_S-induced relaxation using NaHS (10^−9^ – 10^−5^ M) on PE precontracted arteries.

To assess the involvement of S-sulfhydration in H_2_S-induced relaxation, the arterial tissues were preindubated with dithiothreitol (DTT, 2 mM) for 20 min, and then relaxation response to NaHS (10^−9^ – 10^−5^ M) was carried out on PE pre-constricted arteries.

### Statistical analysis

Analyses were performed using Prism (GraphPad 9, San Diego, CA, USA). Cumulative relaxation-response curves were fitted to a 4-parameter sigmoid curve for vascular reactivity. Relaxant responses were expressed as a percentage of relaxation from the PE-induced contraction. The data were presented as the mean ± standard error of the mean (SEM). Statistical significance was considered at a p-value of less than 0.05.

## Results

### H_2_S-induced vascular smooth muscle relaxation response in mesenteric artery and uterine artery from virgin and pregnant rat

**Figure 1:**
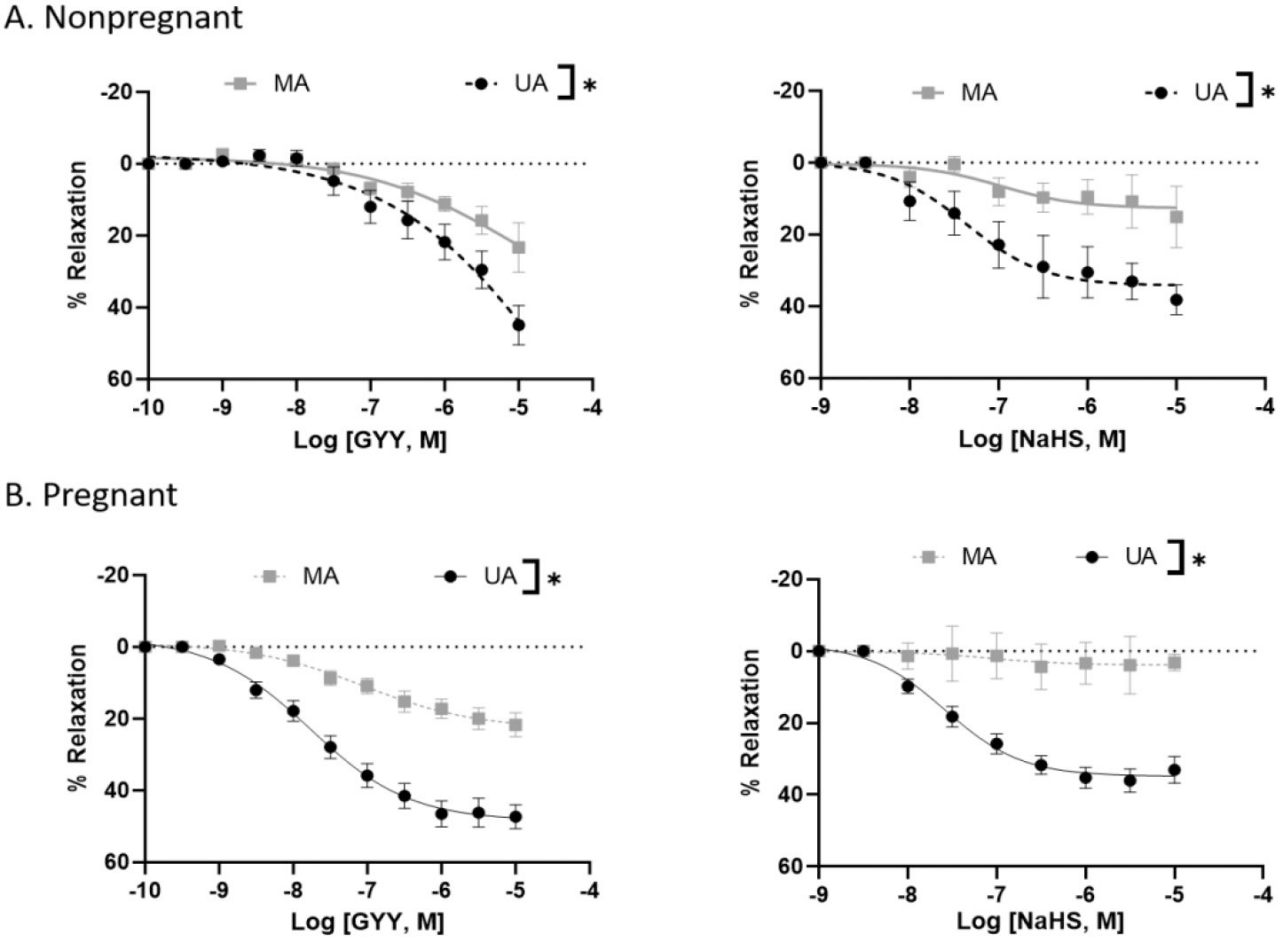
GYY4137 and NaHS-induced concentration-dependent relaxation responses in nonpregnant and pregnant rat mesenteric and uterine arterial rings pre-constricted with PE. The vertical bar represents mean ± S.E.M. (n= 6, *p < 0.05 UA vs. MA). MA – Mesenteric artery; UA – Uterine artery.

In endothelium-denuded arterial rings isolated from nonpregnant rats and precontracted with PE, maximum relaxation to GYY4137 and NaHS was greater in the uterine artery versus the mesenteric artery (Figure 1A and Table 1). Similarly, in the rings of pregnant rats, GYY4137 and NaHS were more potent in causing relaxation in the uterine artery than in the mesenteric artery (Figure 1B and Table 1). The relaxation to GYY4137 and NaHS was not different in the mesenteric artery of pregnant versus virgin rats but was greater in the uterine artery of pregnant versus nonpregnant rats, indicating an enhanced sensitivity of uterine artery to H_2_S-induced relaxation during pregnancy (Figure 1 and Table 1).

### Role of high K concentration on H_2_S-induced relaxation of pregnant uterine arteries

**Figure 2:**
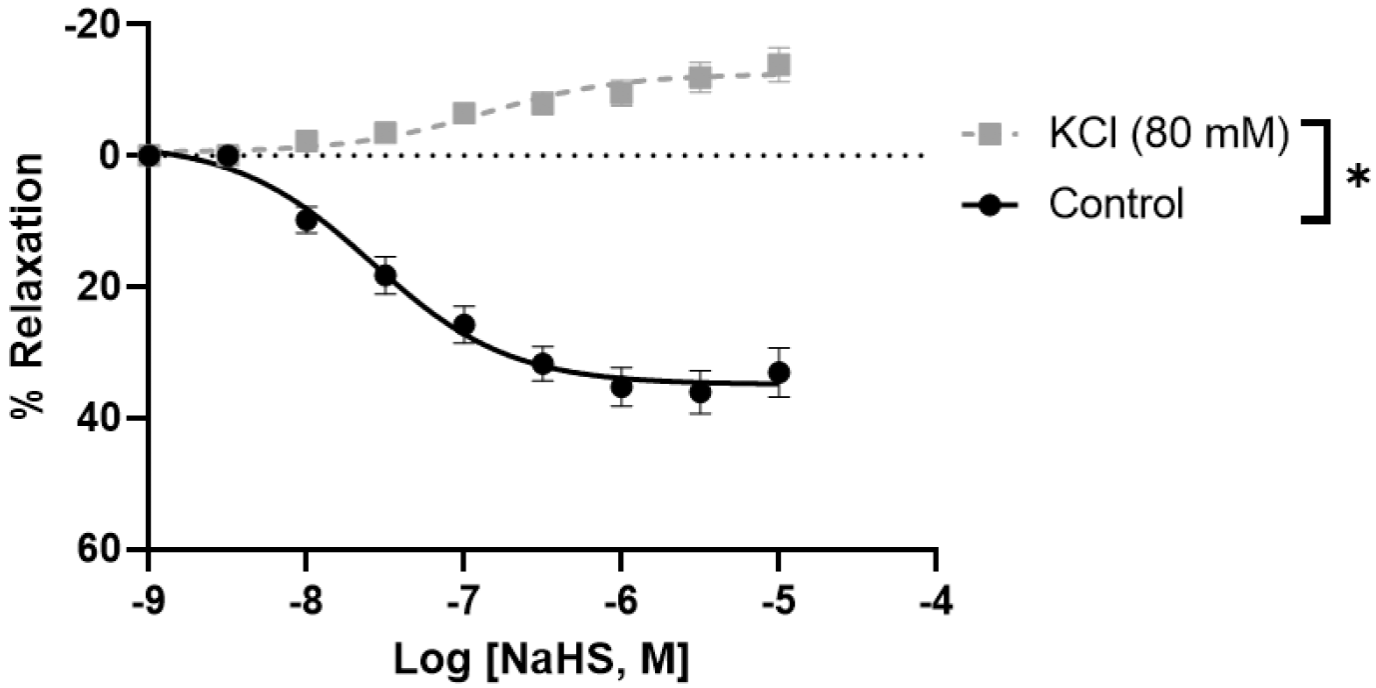
NaHS-induced concentration-dependent relaxation in pregnant rat uterine arterial rings in the presence and absence of high extracellular potassium concentration (80 mM). The vertical bar represents mean ± S.E.M. (n= 6, *p < 0.05 compared to control).

Because the uterine arteries of pregnant rats showed greater vascular relaxation than those of nonpregnant rats, we investigated the mechanisms of H_2_S relaxation in these arterial rings. A high K^+^ concentration (80 mM), which inhibits the opening of K channels involved in smooth muscle relaxation [27], completely blocked the relaxation response to NaHS and shifted the H_2_S response to slight vasoconstriction (Figure 2).

### Role of specific potassium channels on H_2_S-induced relaxation of pregnant uterine arteries

**Figure 3:**
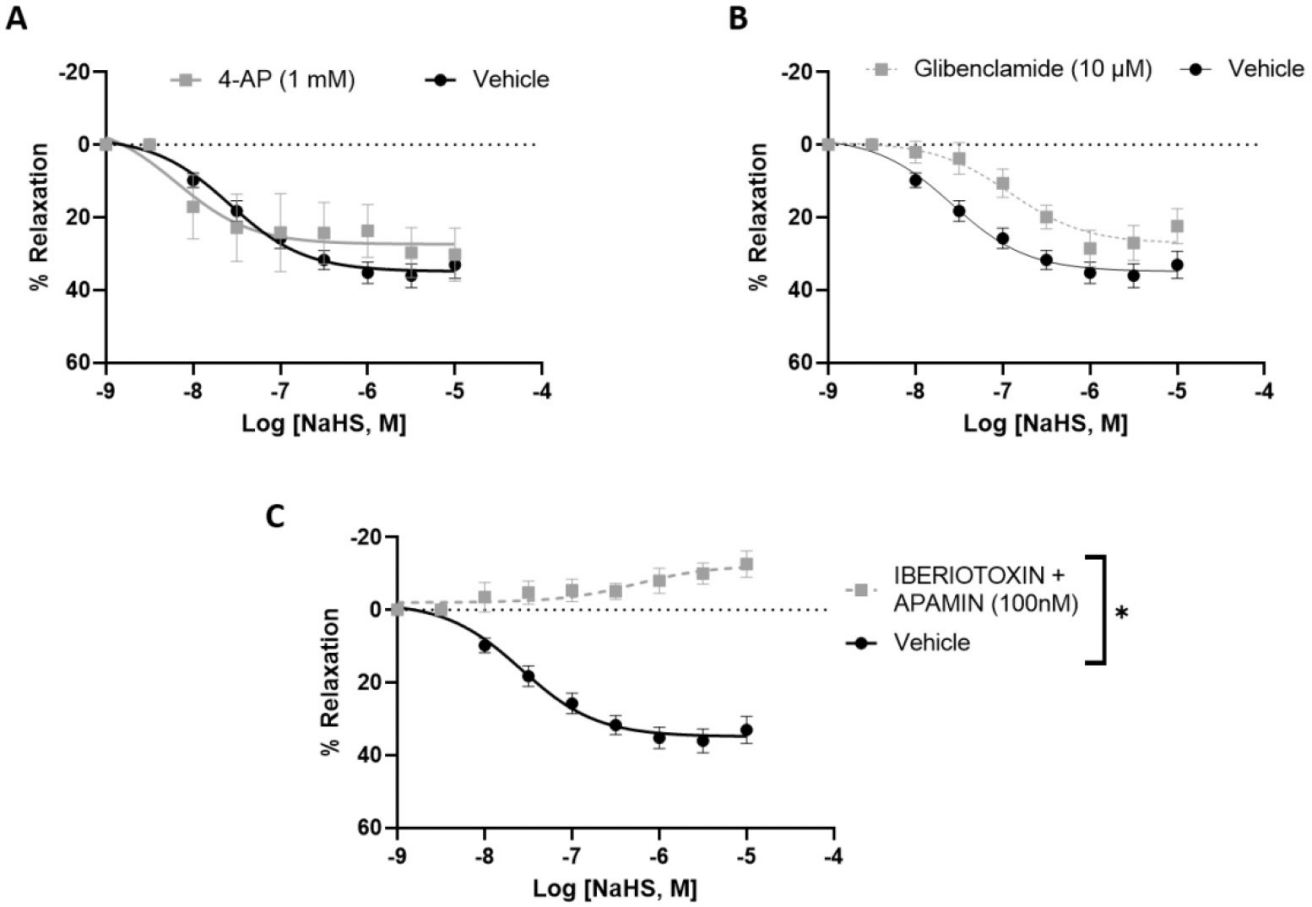
NaHS-induced concentration-dependent relaxation in pregnant rat uterine arterial rings pre-incubated with (A) 4-aminopyridine (1 mM), (B) Glibenclamide (10 μM) and (C) iberiotoxin (100 nM) plus apamin (100 nM) for 20 min and then pre-constricted with PE. The vertical bar represents mean ± S.E.M. (n= 6, *p < 0.05 compared to control).

The involvement of different potassium channels in the H_2_S-mediated relaxation of uterine arteries of pregnant rats was examined by using specific blockers. Blockade of the voltage-gated potassium channel with 4-AP (1 mM) did not affect the H_2_S-induced relaxation response (Figure 3A). Glibenclamide (10 μM) partially reduced the relaxation response of NaHS, suggesting that ATP-sensitive potassium channels have minimal contribution to H_2_S relaxation (Figure 3B). Iberiotoxin (100 nM) plus apamin (100 nM) completely abolished the relaxation response to NaHS (10^−9^ - 10^−5^ M) (Figure 3C), indicating that BK_ca_ channels were essential for H_2_S relaxation. These results demonstrate that H_2_S-induced relaxation of uterine arteries of pregnant rats is mediated by different types of potassium channels, especially BK_Ca_ channels (Figure 3 and Table 2).

### Effect of DTT on H_2_S-induced relaxation of pregnant uterine arteries

**Figure 4:**
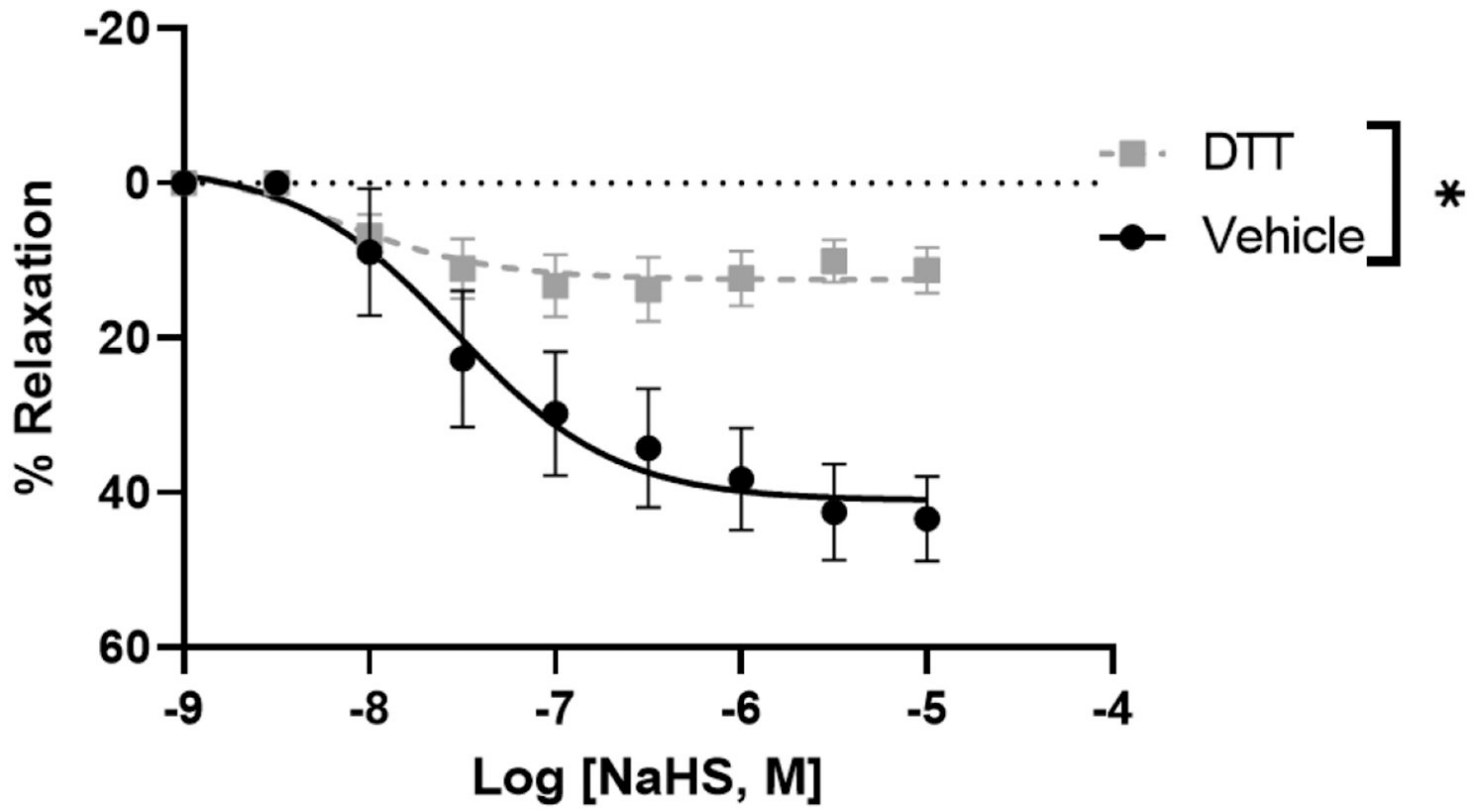
NaHS-induced concentration-dependent relaxation in pregnant rat uterine arterial rings was pre-incubated with dithiothreitol (DTT, 2 mM) for 20 min and then pre-constricted with PE. The vertical bar represents mean ± S.E.M. (n= 6, *p < 0.05 compared to control).

H_2_S exerts its biological effects by S-sulfhydrating cysteine residues on target proteins. Dithiothreitol (DTT) is a reducing agent that can inhibit S-sulfhydration. To test whether S-sulfhydration is involved in the relaxation response of uterine arteries to H_2_S, the arteries were preincubated with DTT (2 mM). DTT significantly decreased the relaxation response of NaHS from 43.38 ± 5.39% to 11.22 ± 2.88% (Figure 4), suggesting that blockade of S-sulfhydration activity and reduces H_2_S-induced vasodilation.

## Discussion

The major findings of the study are, First, GYY4137 and NaHS evoked a concentration-dependent endothelium-independent vasorelaxation that was more pronounced in the uterine than mesenteric arteries. Second, high extracellular K^+^ concentrations abolished the NaHS-induced vasodilation of pregnant rat uterine arteries. Third, NaHS-induced relaxation in pregnant uterine arteries involved activation of K_ATP_ and BK_Ca_ channels. Fourth, suppression of S-sulfhydration reduced the relaxation of pregnant rat uterine arteries to NaHS. Our results demonstrated a vascular bed and pregnancy-specific relaxant response of arteries to H_2_S, with uterine arteries being more sensitive to H_2_S-induced relaxation during pregnancy. Stimulating K_ATP_ and BK_Ca_ channels and S-sulfhydration in vascular SMCs by H_2_S represent important cellular mechanisms for the H_2_S effect in the uterine artery during pregnancy. These data, together with previous studies in which blockade of H_2_S synthesis decreased pup weight and increased pup mortality in rats [28], suggest that the H_2_S may play an important role in maintaining relaxation of the uterine vascular bed and, therefore, in uterine blood flow in rats during pregnancy.

In this study, GYY4137 and NaHS induced only minimal relaxation of the vascular smooth muscle of mesenteric arteries. This is in contrast to prior studies, which demonstrated significant relaxation in mesenteric arteries when exposed to H_2_S [29-31]. The disparity in these findings may be attributed to differences in the mesenteric artery preparations utilized; our study employed endothelium-denuded rings, while earlier studies utilized endothelium-intact rings. The presence of endothelium appears to play a crucial role in H_2_S-induced relaxation in the mesenteric artery, as indicated by previous studies showing reduced H_2_S relaxations in preparations lacking endothelium in small mesenteric arteries and in the presence of an inhibitor of NO synthase [32]. This implies that endothelium-derived NO is involved in H_2_S relaxations in mesenteric arteries. Our study also provides evidence that H_2_S-induced relaxation in mesenteric arteries was not different between arteries from nonpregnant and pregnant rats, indicating that pregnancy does not influence H_2_S-induced mesenteric vasodilation during pregnancy. Thus, these findings suggest that H_2_S exerts only minimal direct vascular smooth muscle relaxation effects, and this effect remains unaltered by pregnancy. It would be interesting to examine whether the sensitivity of H_2_S vasodilation is affected by pregnancy in endothelium-intact mesenteric arteries.

On the other hand, H_2_S induced greater relaxation in the uterine than mesenteric arteries. Studies show that the uterine artery relaxes more than the mesenteric artery during pregnancy. This is because the uterine artery needs to accommodate the increased blood flow to the placenta and the fetus, while the mesenteric artery does not have such a demand. One study found that the uterine artery resistance index, which is a measure of vascular resistance, decreased significantly from early to late gestation, while the mesenteric artery resistance index did not change much [33]. Another study reported that the uterine artery pulsatility index, which is another measure of vascular resistance, decreased over gestation, while the mesenteric artery pulsatility index increased slightly [34]. These findings suggest that the uterine artery relaxes more than the mesenteric artery during pregnancy to facilitate placental perfusion and fetal growth. However, the mechanisms contributing to the enhanced vasodilation of the pregnant uterine arteries were unclear. Our finding that H_2_S produces greater relaxation in the uterine than mesenteric arteries and that the relaxation effect in the uterine arteries is further enhanced during pregnancy suggests that H_2_S could be an important factor in supporting gestational uterine vascular adaptations.

Various mechanisms have been proposed to elucidate the vasodilatory effects of H_2_S, depending on the specific vascular preparations under investigation. Previous studies focusing on resistance arteries have identified the involvement of K_ATP_ channels [16], BK_Ca_ channels [18, 35], and K_V_ channels [29, 36] in H_2_S-induced vasodilation. Patch-clamp studies have demonstrated that H_2_S activates K_ATP_ channels in vascular smooth muscle from mesenteric arteries [16] and hyperpolarizes mesenteric arteries through an iberiotoxin-sensitive mechanism, indicating the participation of BK_Ca_ channels [18, 35]. In separate experiments, H_2_S was found to hyperpolarize rat aorta and directly activate K_V_ channels in CHO cells [37]. Recent investigations have revealed that H_2_S donors directly activate K_V_ channels, providing protection against neuropathic pain [38]. Additionally, direct persulfidation of K_V_ channels by H_2_S has been implicated in skeletal muscle hypercontractility in human malignant hyperthermia syndrome [39]. In alignment with findings in pregnant uterine arteries, our study indicates that H_2_S-induced relaxations are sensitive to high extracellular potassium and are blocked by inhibitors of both ATP-sensitive and BK_Ca_ channels. This suggests the involvement of K_ATP_ and BK_Ca_ channels in H_2_S-mediated relaxation. Intriguingly, the presence of a K_ATP_ channel blocker has a lesser impact on the magnitude of H_2_S-induced relaxation compared to BK_Ca_ channel blockers, implying a predominant role of BK_Ca_ channels in mediating H_2_S-induced relaxation in pregnant rat uterine arteries. This aligns with prior research demonstrating that H_2_S enhances BK_Ca_ currents in a dose-dependent manner, leading to the relaxation of PE pre-constricted human uterine arterial rings, which was inhibited by the BK_Ca_ channel blocker [19].

Although our findings do not elucidate the mechanism by which H_2_S modulates BK_Ca_ channel activity, at least 2 possibilities exist. Firstly, BK channels are known to be regulated by zinc (Zn) through coordination with histidine and aspartic acid residues in the RCK1 (regulator of conductance for K^+^) domain of the channel [40]. Removal of Zn reduces channel activation, suggesting a potential structural arrangement for Zn binding by these residues [40]. Concurrently, H_2_S has been identified as a binder of Zn, capable of altering the activity of Zn-dependent proteins [41]. A second potential mechanism through which H_2_S may regulate BK_Ca_ channel activity is sulfhydration. Recent mass spectrometric analyses have unveiled the addition of extra sulfur to thiol (-SH) groups of cysteines, forming a hydropersulfide (-SSH) moiety [42]. This posttranslational modification affects a subset of proteins, including but not limited to GAPDH, β-tubulin, and actin, altering their functions [43]. Notably, our observation that inhibition of sulfhydration with DTT diminishes H_2_S-induced relaxation supports the hypothesis that H_2_S modifies critical cysteine residues regulating BK channel activity. This aligns with previous bioinformatics studies identifying pregnancy-dependent H_2_S-responsive human uterine artery SSH peptides/proteins, particularly those involved in vascular smooth muscle contraction/relaxation [44]. However, further verification is necessary to confirm whether H_2_S regulates the sulfhydration of BK_Ca_ channels and if this effect is heightened to induce more substantial vasodilation in pregnant rat uterine arteries.

In conclusion, H_2_S exhibit regional changes in their vasodilator activity in the vascular smooth muscle of the mesenteric artery and uterine artery. The H_2_S-induced vasodilator effect is enhanced in the uterine artery during gestation. The data also support the premise that H_2_S-mediated uterine vasodilatation may be mediated more by BK_Ca_ channels and sulfhydration. The study supports the proposition that pregnancy-associated changes in vascular H_2_S activity in the uterine versus systemic circulation could regulate uterine blood flow and distribution of blood in the fetoplacental unit by promoting vasodilation during normal pregnancy. Future studies should investigate the potential usefulness of H_2_S donors in promoting vasodilation and improving maternal hemodynamics and uterine artery blood flow in preeclamptic pregnancies.

## Figures and Tables

**Table 1:** GYY4137 and NaHS-induced relaxation of the mesenteric and uterine arteries in nonpregnant and pregnant rats.

	Nonpregnant		Pregnant	
	MA	UA	MA	UA
**GYY4137-induced relaxation**				
Maximal relaxation	23.28 ± 6.81	44.88 ± 5.35*	21.70 ± 3.27	47.30 ± 3.28*
**NaHS-induced relaxation**				
Maximal relaxation	15.11 ± 8.65	38.14 ± 4.07*	3.19 ± 1.48	36.03 ± 3.22*

* p < 0.05 UA vs MA in respective groups (n=6 in each group). MA – Mesenteric artery; UA – Uterine artery

**Table 2:** NaHS-induced response in the presence and absence of K^+^ channel blockers and KCl

Pregnant Uterine arteries	Control	4-AP	Glibenclamide	Iberiotoxin and apamin
Maximal relaxation	36.03 ± 3.22	30.22 ± 7.21	28.55 ± 4.94*	−8.03 ± 3.77*

* p < 0.05 compared to control (n = 6 in each group).
